# Seroprevalence of severe acute respiratory coronavirus virus 2 (SARS-CoV-2) antibodies among healthcare workers with differing levels of coronavirus disease 2019 (COVID-19) patient exposure

**DOI:** 10.1017/ice.2020.390

**Published:** 2020-08-03

**Authors:** Benton R. Hunter, Lana Dbeibo, Christopher S. Weaver, Cole Beeler, Michele Saysana, Michelle K. Zimmerman, Lindsay Weaver

**Affiliations:** 1 Department of Emergency Medicine, Indiana University School of Medicine Indianapolis, Indiana; 2 Division of Infectious Diseases, Department of Internal Medicine, Indiana University School of Medicine, Indianapolis, Indiana; 3 Indiana University Health, Indianapolis, Indiana; 4 Department of Pediatrics, Indiana University School of Medicine, Indianapolis, Indiana; 5 Department of Pathology and Laboratory Medicine, Indiana University School of Medicine, Indianapolis, Indiana

## Abstract

Healthcare employees were tested for antibodies against severe acute respiratory coronavirus virus 2 (SARS-CoV-2). Among 734 employees, the prevalence of SARS-CoV-2 antibodies was 1.6%. Employees with heavy coronavirus disease 2019 (COVID-19) exposure had similar antibody prevalence as those with limited or no exposure. Guidelines for PPE use seem effective for preventing COVID-19 infection in healthcare workers.

Concern and disagreement have arisen regarding what personal protective equipment (PPE) is required to safely protect frontline healthcare workers (HCWs) from severe acute respiratory coronavirus 2 (SARS-CoV-2).^1,2^ Recommendations have changed and angst has been fueled by a lack of respirators and other PPE in several countries.^1,3^ Frontline HCWs in COVID-19 units may have a higher rate of infection than those in the general population.^4^ Our institution follows the World Health Organization (WHO) guidelines for PPE in the COVID-19 pandemic, including universal masking, with respirator (N95 or equivalent) utilization only during approved aerosol-generating procedures.^5^ The seroconversion rate among frontline providers could provide valuable information regarding the effectiveness of our PPE strategy.

The goal of this investigation was to compare the proportion of people with SARS-CoV-2 antibodies among a large group of HCWs with differing levels of exposure to patients with COVID-19. We also sought to compare the prevalence of confirmed COVID-19 in HCWs with that of the general population in the state, which at the time was ~2.8%.^6^

## Methods

This study was granted approval under exempt status by the local institutional review board.

### Participants and settings

Indiana University Health is a large, integrated healthcare system with 17 hospitals and ~35,000 employees across the state of Indiana. We deployed 760 antibody tests to employees from 18 different locations, including 14 hospitals, 3 outpatient centers, and an administrative building where employees have no patient exposure. Hospitals were categorized as high risk if they had admitted a total of at least 30 confirmed cases of COVID-19, and low risk if they had admitted <30. Of 14 hospitals, 6 were deemed high risk.

Open invitations to volunteer for serology testing were sent via e-mail to groups of employees at each setting. For healthcare settings with direct patient contact, the number of tests allocated to each setting was stratified by patient volume. Employees with active symptoms or previously confirmed COVID-19 were excluded. At clinical sites, all providers, nurses, and respiratory therapists were invited to participate. Since we were testing for IgG, asymptomatic employees who tested positive were not asked to quarantine or take off work. After invitations were sent, volunteers responded by e-mail and were accepted on a first-come–first-serve basis until the tests allocated to that setting were all accounted for. For hospital settings, invitations were only extended to groups of employees with high-risk patient exposure: intensive care unit (ICU), ED, or hospitalist physicians, advanced practice providers, nurses, and respiratory therapists. For high prevalence settings, serology testing was only offered to those working in the ED or on COVID units.

**Table 1.** Results by Employee Title

Characteristic	Positive (n = 12)	Negative (n = 722)	Total (n = 734)
Age, mean y	44.5	42.7	42.8
Female sex, %	75	70	70
Physician/APP	3	276	279
Registered nurse	7	310	317
Respiratory therapist	0	94	94
Administrator	2	42	44
Total	12	722	734

### Testing

All antibody testing was performed on serum samples using the Emergency Use Authorization granted Abbott Architect i2000SR chemiluminescent microparticle immunoassay for the qualitative detection of SARS-CoV-2 IgG antibody. This test was approved by the Food and Drug Administration based on data showing 99.6% specificity and 100% sensitivity for patients with confirmed COVID-19 who had >14 days of symptoms. Serology testing was performed at a single centralized laboratory between April 29, 2020, and May 8, 2020.

### Statistical analysis

Seroprevalence was stratified by high- versus low-risk work environment, and healthcare role (ie, nurse, administrator, physician/provider, or respiratory therapist). Comparative rates are reported as relative risk (RR) with 95% confidence intervals (CIs), calculated using a Taylor series. The preplanned comparisons were high-risk versus low-risk setting, nurses (thought to be highest risk secondary to spending the most time with direct patient contact) versus all others, and administration (no patient contact) versus all others.

## Results

Of 760 individuals who volunteered for antibody testing, 734 (96.6%) completed the study. The average age was 43 years and 70.1% were female. Moreover, 12 employees (1.6%) tested positive for SARS-CoV-2 IgG antibodies. Employees from high-risk settings comprised 52.5% of those tested and had a 1.3% positive rate (5 of 385), compared to 2.0% in low-risk employees (7 of 349). The difference in rates was not statistically significant (relative risk [RR], 0.65; 95% CI, 0.21–2.0). Nurses had a similar rate of positive cases to other employees (2.2% vs 1.2%; RR, 1.9; 95% CI, 0.60–5.8). Of 44 administrative staff with no patient contact, 2 were positive, for a rate of 4.5%, which was not statistically significantly different than patient facing employees (RR, 3.1; 95% CI, 0.71–13.9). Table 1 displays demographics and stratified testing results.

## Discussion

We found a low rate of antibodies to SARS-CoV-2 among a large group of healthcare employees, including those with exposure to a large number of COVID-19 patients. Furthermore, HCWs did not appear to be at higher risk than employees with no patient contact or the general population. At the time our study was being carried out, the Indiana State Health Department performed serologic and PCR testing across the state. Among 4,000 randomly selected volunteers, they found a prevalence of 2.8%,^6^ similar to our population of largely high-risk HCWs. Workers with previously confirmed COVID-19 infection were excluded from our study. However, at the time that we began the study, only 1.3% of total employees had tested positive for COVID-19. Adding this to our seroprevalence yields a 2.9% infection rate, almost identical to that found in the general population in Indiana.

Perhaps the most interesting finding in our study was that HCWs with the most exposure to COVID-19 patients were not at higher risk for developing antibodies than employees with little to no work-related COVID-19 exposure. Other studies have suggested front line HCWs are more likely to have antibodies or test positive for active COVID-19 than the general population.^4^ This may be attributable to our institutional application of WHO guidelines for PPE use.^5^ Respirators (N-95) were utilized only for aerosolizing procedures (eg, intubation, use of nebulizers, bronchoscopy) to conserve high-level PPE. Early in the pandemic, the prevalence of SARS-CoV-2 was much lower in the state than in other areas with more dense populations that were hit earlier, such as Seattle, Los Angeles, and New York City. Thus, our hospital system had additional time to prepare, which also may have contributed to a low infection rate among HCWs.

This cross-sectional study has several limitations. Employees were included based on volunteering to participate. Volunteers may have had more reason to think they could be positive than those who did not volunteer. Although the overall number of people tested was, to our knowledge, the largest group of HCWs in a single study to date, the number of administrative employees (those with no patient contact) was small (n = 44), resulting in large confidence intervals around the relative risk for them compared to frontline HCWs. At the time this study was conducted, our hospital system had seen ~1,000 confirmed COVID-19 patients, representing a very different environment than that seen in very hard hit areas such as New York City. Our results may not extrapolate to settings with overwhelming numbers of community COVID-19 infections.

In conclusion, we found a low prevalence of seroconversion to indicate previous SARS-CoV-2 infection among a large group of HCWs without a pre-existing diagnosis of COVID-19. Frontline HCWs with heavy exposure to infected patients did not have higher rates of seroconversion than employees with little or no patient exposure.
